# Functional respiratory imaging assessment of budesonide/glycopyrrolate/formoterol fumarate and glycopyrrolate/formoterol fumarate metered dose inhalers in patients with COPD: the value of inhaled corticosteroids

**DOI:** 10.1186/s12931-021-01772-2

**Published:** 2021-07-01

**Authors:** Maarten van den Berge, Jan De Backer, Cedric Van Holsbeke, Wilfried De Backer, Roopa Trivedi, Martin Jenkins, Paul Dorinsky, Magnus Aurivillius

**Affiliations:** 1 Department of Pulmonary Diseases, Groningen Research Institute for Asthma and COPD (GRIAC), University of Groningen, University Medical Center Groningen, Groningen, The Netherlands; 2 FLUIDDA Inc, Los Angeles, CA USA; 3 University of Antwerp, Antwerp, Belgium; 4 AstraZeneca, Durham, NC USA; 5 AstraZeneca, Cambridge, UK; 6 AstraZeneca, Gothenburg, Sweden

**Keywords:** Budesonide, COPD, Formoterol fumarate, Functional respiratory imaging, Glycopyrrolate, Triple therapy

## Abstract

**Background:**

For patients with chronic obstructive pulmonary disease (COPD), greater improvements in lung function have been demonstrated for triple versus dual inhaled therapies in traditional spirometry studies. This study was the first to use functional respiratory imaging (FRI), known for increased sensitivity to airway changes versus spirometry, to assess the effect of the inhaled corticosteroid (ICS) component (budesonide) on lung function in patients with moderate-to-severe COPD and a blood eosinophil count > 150 cells/mm^3^.

**Methods:**

Patients in this Phase IIIb (NCT03836677), randomized, double-blind, crossover study received twice-daily budesonide/glycopyrrolate/formoterol fumarate (BGF) 320/18/9.6 μg fixed-dose triple therapy and glycopyrrolate/formoterol fumarate (GFF) 18/9.6 μg fixed-dose dual therapy over 4 weeks, each delivered via a single metered dose Aerosphere inhaler. Primary endpoints were the improvements from baseline for each treatment in specific (i.e. corrected for lobar volume) image-based airway volume (siVaw) and resistance (siRaw) measured via FRI taken at total lung capacity (Day 29). Secondary outcomes included spirometry and body plethysmography. Adverse events were monitored throughout the study.

**Results:**

A total of 23 patients were randomized and included in the intent-to-treat analysis (mean age 64.9 years, 78.3% males, 43.5% current smokers, mean predicted post-bronchodilator forced expiratory volume in 1 s [FEV_1_] 63.6%). BGF and GFF both statistically significantly increased siVaw from baseline at Day 29 (geometric mean ratio [GM], 95% confidence interval [CI]: 1.72 [1.38, 2.13] and 1.53 [1.28, 1.83], respectively, both *p* < 0.0001), with a greater increase observed for BGF versus GFF (GM, 95% CI 1.09 [1.03, 1.16], *p* = 0.0061). Statistically significant reductions in siRaw were also observed with both BGF and GFF (GM, 95% CI 0.50 [0.39, 0.63] and 0.52 [0.40, 0.67], respectively, both *p* < 0.0001). Additionally, significant improvements from baseline in post-dose FEV_1_ were observed with BGF and GFF (mean 346 mL, *p* = 0.0003 and 273 mL, *p* = 0.0004, respectively). Safety findings were consistent with the known profiles of BGF and GFF.

**Conclusions:**

As observed using FRI, triple therapy with BGF resulted in greater increases in airway volume, and reductions in airway resistance versus long-acting muscarinic antagonist/long-acting β_2_-agonist (LAMA/LABA) dual therapy with GFF, reflecting the ICS component’s contribution in patients with moderate-to-severe COPD.

*Trial registration:* ClinicalTrials.gov, NCT03836677. Registered 11 February 2019, https://clinicaltrials.gov/ct2/show/NCT03836677

**Supplementary Information:**

The online version contains supplementary material available at 10.1186/s12931-021-01772-2.

## Background

Triple therapy using inhaled corticosteroids (ICS), long-acting β_2_-agonists (LABA) and long-acting muscarinic antagonists (LAMA) is recommended for the treatment of patients with chronic obstructive pulmonary disease (COPD) and continued symptoms or exacerbations despite dual therapy with an ICS/LABA or LAMA/LABA [1].

The triple fixed-dose combination budesonide/glycopyrrolate/formoterol fumarate (BGF) metered dose inhaler (MDI) was shown to improve symptoms, improve lung function and reduce exacerbation rates versus glycopyrrolate/formoterol fumarate (GFF) MDI and budesonide/formoterol fumarate (BFF) MDI in symptomatic patients with COPD in the ETHOS (NCT02465567) and KRONOS (NCT02497001) studies [2, 3]. In addition, BGF was shown to be efficiently deposited throughout the lung in scintigraphy studies in healthy subjects [4] and in patients with moderate-to-very severe COPD [5].

Functional respiratory imaging (FRI; US Food and Drug Administration clearance for use in clinical practice received in March 2020 [K191550] [6]) is a computed tomography (CT)-based, quantitative post-processing technology that can be used to assess parameters of airway volume and airway resistance to resolutions of 0.23–0.35 mm [7, 8]. Traditional spirometric assessments do not assess local, region-specific airway changes following treatment, and in several previous studies FRI has proven to be more sensitive than spirometric assessments [9–11]. In this regard, previous studies of bronchodilators have used FRI to show local, region-specific airway changes post-treatment, including the LAMA/LABA combination GFF, relative to placebo in patients with stable COPD [9, 10, 12, 13]. Furthermore, both the glycopyrrolate and formoterol fumarate monocomponents of GFF significantly improved FRI parameters versus baseline in patients with COPD [12]. To date, FRI has not been used to assess the effect of the ICS component in patients with COPD.

Here we report the results of study NCT03836677 (D5980C00019), the first study to utilize FRI to assess the effect of the ICS budesonide by evaluating fixed-dose triple therapy with BGF and fixed-dose dual LAMA/LABA therapy GFF in patients with moderate-to-severe COPD. To assess this, data from FRI parameters, spirometry, and body plethysmography were analyzed to determine the effects of BGF versus GFF on improving specific image-based volume (siVaw) and resistance (siRaw).

## Methods and materials

### Study design

This randomized, double-blind, Phase IIIb, crossover study (NCT03836677) evaluated and compared the effects of BGF (320/18/9.6 μg) with the effects of GFF (18/9.6 μg) over 4 weeks on FRI parameters, as well as spirometric and plethysmographic parameters, in patients with moderate-to-severe COPD.

**Fig. 1:**
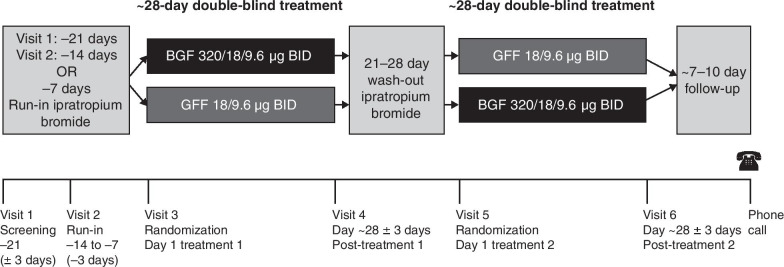
Study design. *BGF* budesonide/glycopyrrolate/formoterol fumarate, *BID* twice-daily, *GFF* glycopyrrolate/formoterol fumarate

Between February 26, 2019 and November 11, 2019, patients were randomized into one of two treatment sequences: BGF followed by GFF, or GFF followed by BGF (Fig. 1). Each dose is expressed as the sum of two actuations given twice-daily via a single Aerosphere inhaler. Doses are expressed as glycopyrrolate 18 μg and formoterol fumarate 9.6 μg, equivalent to glycopyrronium 14.4 μg and formoterol fumarate dihydrate 10 μg, respectively. Patients received approximately 4 weeks of each study treatment, separated by a washout period of 21 to 28 days with treatment of ipratropium bromide (Atrovent hydrofluoroalkane). Ipratropium bromide was also used in the run-in periods of the study.

High-resolution computed tomography (HRCT) scans were performed on Day 1 of period 1 (baseline) and on Day 29 of each treatment period, at total lung capacity (TLC) and functional residual capacity (FRC). An additional CT scan of the upper airway was taken on Day 1 of treatment period 1 (Visit 3) while breathing through the mouthpiece during normal slow inhalation, without a nose clip. The scan of the upper airway was used to reliably assess thoracic deposition of GFF and BGF, as upper airway geometry is a key determinant of lower airway deposition. Spirometric assessments occurred at all visits. At Visit 1, pre- and post-dose; at Visit 3 and 5, pre-dose only; and at Visit 4 and Visit 6 (Day 29 ± 3 days in each treatment period), spirometric parameters were measured pre- and post-dose.

Participants signed an informed consent form, approved by the Independent Ethics Committee and sponsor before study initiation. This study was performed in accordance with the ethical principles that have their origin in the Declaration of Helsinki and that are consistent with International Council for Harmonisation/Good Clinical Practice and applicable regulatory requirements.

### Study participants

Eligible patients were 40–80 years of age; current or former smokers with ≥ 10 pack-years smoking history; had an established diagnosis of COPD as defined by American Thoracic Society/European Respiratory Society criteria [14]; had moderate-to-severe COPD, defined as a forced expiratory volume in 1 s [FEV_1_]/forced vital capacity [FVC] ratio of < 0.70 and a post-bronchodilator FEV_1_ > 30% and < 80% predicted at Visit 2; and had blood eosinophil count > 150 cells/mm^3^ at Visit 1. The cut-off of > 150 cells/mm^3^ was the same as that used as a level to stratify randomization in the KRONOS [3] and ETHOS [2] studies, since lung function benefits of ICS are known to increase with eosinophil count [1]. Patients were on ≥ 1 scheduled maintenance bronchodilator and had no ICS use in the 3 months prior to screening (Visit 1).

Exclusion criteria included a diagnosis of any clinically significant disease other than COPD (inclusive of asthma). In addition, patients with poorly controlled COPD, identified by acute worsening of COPD requiring oral corticosteroid treatment and/or antibiotics in the 3 months prior to Visit 1, or during the run-in period (Visit 1–Visit 3) were excluded.

### Assessments

The primary FRI endpoints were change from baseline in specific (i.e. corrected for lobar volume) siVaw and siRaw. Secondary endpoints were image-based airway volume (iVaw) and resistance (iRaw), as well as FEV_1_ and FRC measured using spirometry and body plethysmography, respectively. Other spirometric endpoints, forced expiratory flow 25–75% [FEF_25–75_] and FVC, were also measured in accordance with previously set criteria [15].

All endpoints were based on change from baseline post-dose assessments performed within 150 min of dosing on Day 29 (± 3 days), with HRCT scans performed 90 min ± 30 min post-dose, followed by spirometry and body plethysmography. Baseline FRI measurements were recorded on Day 1, at least 30 min pre-dose. In order to reduce patient radiation exposure, only one scan was taken at baseline, as treatment is not expected to influence extrathoracic geometry. Adverse events (AEs) were monitored throughout the study.

In addition, we analyzed the mass of deposited particles using FRI methodology [16]. Calculated using computational fluid dynamics, this technique simulates airflow inside a three-dimensional model of patient-specific airways, and how drug particles will travel throughout the respiratory system.

### Statistical analyses

Sample size was chosen based on two previously published FRI studies in similar populations [12, 13], each of which included approximately 20 patients. To attain 80% power to demonstrate improvement from baseline after a Hochberg procedure, a 21% increase from baseline in siVaw (ratio to baseline of 1.213) and 38% reduction from baseline in siRaw (ratio to baseline of 0.621) would be required assuming log-scale standard deviations of 0.262 for siVaw and 0.644 for siRaw. These ratios are similar to those achieved by formoterol monotherapy in a previous study [12]. Hence, it was assumed that the combination treatments used in this study, each containing formoterol, would achieve at least this effect.

The intent-to-treat (ITT) population was defined as all patients who were randomized to treatment. The modified ITT (mITT) population was defined as all patients who completed both treatment periods, having data at baseline and after 4 weeks’ treatment, with data judged to be impacted by important protocol deviations excluded. The safety population included all patients who received ≥ 1 dose of study drug.

FRI parameters used data averaged across all lung lobes, and separately for each lobe to estimate differences in effects between treatments. Baseline was recorded as Day 1 of treatment period 1 (Visit 3).

Primary efficacy analyses (siVaw and siRaw) consisted of a within-treatment comparison of baseline to Day 29 and used a paired t-test in the ITT population. This was performed separately for siVaw and siRaw, within each of the BGF and GFF treatment groups in the ITT population. Both siVaw and siRaw were measured on the conducting airways visible on the HRCT scan. The smallest airways that can be resolved are of 1–2 mm in diameter. When a treatment is administered to dilate the airways, a widening of the lumen will be observed. Additionally, more airway generations will be visible as the HRCT scanner picks up branches that were previously too small. To distinguish between both mechanisms, airway volumes and resistances were assessed both “trimmed” and “untrimmed”. The “untrimmed” analysis included all airway branches visible at a specific visit, while the “trimmed” analysis only included airway branches visible across different visits.

For the primary efficacy endpoints, Hochberg’s step-up procedure was used to adjust for multiplicity. It was applied once for siVaw and siRaw endpoints of BGF, and separately again for GFF. There was no multiplicity adjustment for secondary endpoints or for comparisons of treatments. Two-sided *p*-values were reported to a significance level of 0.05.

Estimates were also produced for the difference between the BGF and GFF treatment groups, by lobe, and across all lobes. A multilevel, by lobe model was used to incorporate the repeated measurements from the lobes for each patient, including fixed effects for period, treatment, lobe, and treatment-by-lobe interaction. Lobe was included as a random effect within each patient. Data were logarithmically transformed before analysis with treatment effect estimates, then exponentiated, and presented as ratios.

The secondary endpoints of iVaw and iRaw were analyzed across lobes similarly to the primary endpoints, with untrimmed data being used, and were not corrected for lobe volume.

For spirometric and plethysmographic endpoints at Day 29, paired tests were compared with assessments at Visit 3 and Visit 5 (Day 1 of each treatment period) with averaging over–60- and -30–min values for spirometry. For comparisons between treatments, a patient-level baseline for a given endpoint was defined as the average of the corresponding period-dependent baselines.

All statistical analyses were performed using SAS 9.4 or other validated software as appropriate.

## Results

### Study population

A total of 23 patients were randomized and received ≥ 1 dose of the study drug, and were included in the ITT and safety analysis populations. Of these, 22 patients completed treatment with BGF, and 21 with GFF. Two patients in the BGF treatment group did not have HRCT scans completed in the BGF treatment periods, therefore 20 patients were included in the primary analysis for BGF, and 21 for GFF. A total of 17 patients (73.9%) were included in the mITT analysis set; four patients (17.4%) reported protocol deviations and two patients (8.7%) did not complete both treatment periods.

Most patients in the study were male (78.3%), and the mean age of patients in the study was 64.9 years; 43.5% were current smokers (Table 1). At Visit 1, 73.9% of patients had moderate COPD and 26.1% had severe COPD. The overall mean (standard deviation, SD) post-bronchodilator FEV_1_ was 63.6% (13.7) of predicted normal (Table 1).

**Table 1:** Baseline demographics and characteristics (ITT population)

	BGF 320/18/9.6 µg (*N* = 22)	GFF 18/9.6 µg (*N* = 23)	Total (*N* = 23)
Mean age, years (SD)	64.8 (7.8)	64.9 (7.6)	64.9 (7.6)
Male, *n* (%)	17 (77.3)	18 (78.3)	18 (78.3)
Current smoker, *n* (%)	10 (45.5)	10 (43.5)	10 (43.5)
Median pack-years smoked, (range)	40.5 (15–100)	41.0 (15–100)	41.0 (15–100)
Severity of COPD (GOLD), *n* (%)			
Moderate	17 (77.0)	17 (73.9)	17 (73.9)
Severe^a^	5 (22.7)	6 (26.1)	6 (26.1)
COPD exacerbations per patient (past 12 months), mean (SD)	0.2 (0.5)	0.2 (0.5)	0.2 (0.5)
Total CAT score (0–40)^b^, mean (SD)	17.6 (5.5)	17.3 (5.6)	17.3 (5.6)
FEV_1_ at Visit 1 (% predicted)			
Pre-bronchodilator, mean (SD)	58.9 (13.3)	58.4 (13.1)	58.4 (13.1)
Post-bronchodilator, mean (SD)	64.1 (13.7)	63.6 (13.7)	63.6 (13.7)
FEV_1_/FRC post-bronchodilator at Visit 1, mean (SD)	52.0 (10.6)	51.7 (10.5)	51.7 (10.5)
% predicted RV, mean (SD)	173.7 (44.9)	173.2 (43.9)	173.2 (43.9)
TLC (L), mean (SD)	7.5 (1.5)	7.4 (1.4)	7.4 (1.4)
% predicted FRC, mean (SD)	150.4 (26.6)	149.8 (26.2)	149.8 (26.2)

^a^One patient was reported with very severe COPD on the electronic case report form; however, all FEV_1_ values at Visit 1 and Visit 3 fell within the inclusion criteria (30–< 80%) and the patient was correctly randomized as having severe COPD

^b^The total score was the sum of 8 CAT item scores. A higher scored denotes more severe impact of COPD

*BGF* budesonide/glycopyrrolate/formoterol fumarate; *CAT* COPD Assessment Test; *COPD* chronic obstructive pulmonary disease; *FEV*_*1*_ forced expiratory volume in 1 s; *FRC* functional residual capacity; *GFF* glycopyrrolate/formoterol fumarate; *GOLD* The Global Initiative for Chronic Obstructive Lung Disease; *ITT* intent-to-treat; *RV* residual volume; *SD* standard deviation; *TLC* total lung capacity

### FRI

**Fig. 2:**
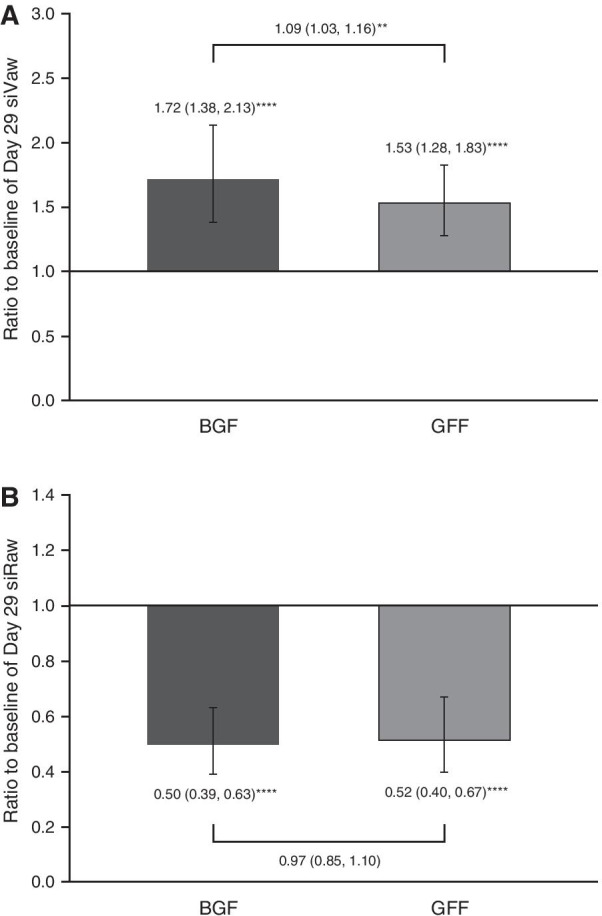
Geometric mean ratio to baseline: **a** siVaw and **b** siRaw at Day 29. ****p ≤ 0.0001, **p < 0.01. Error bars show 95% CI. *BGF* budesonide/glycopyrrolate/formoterol fumarate, *CI* confidence interval, *GFF* glycopyrrolate/formoterol fumarate, *siRaw* specific image-based airway resistance, *siVaw* specific image-based airway volume

**Fig. 3:**
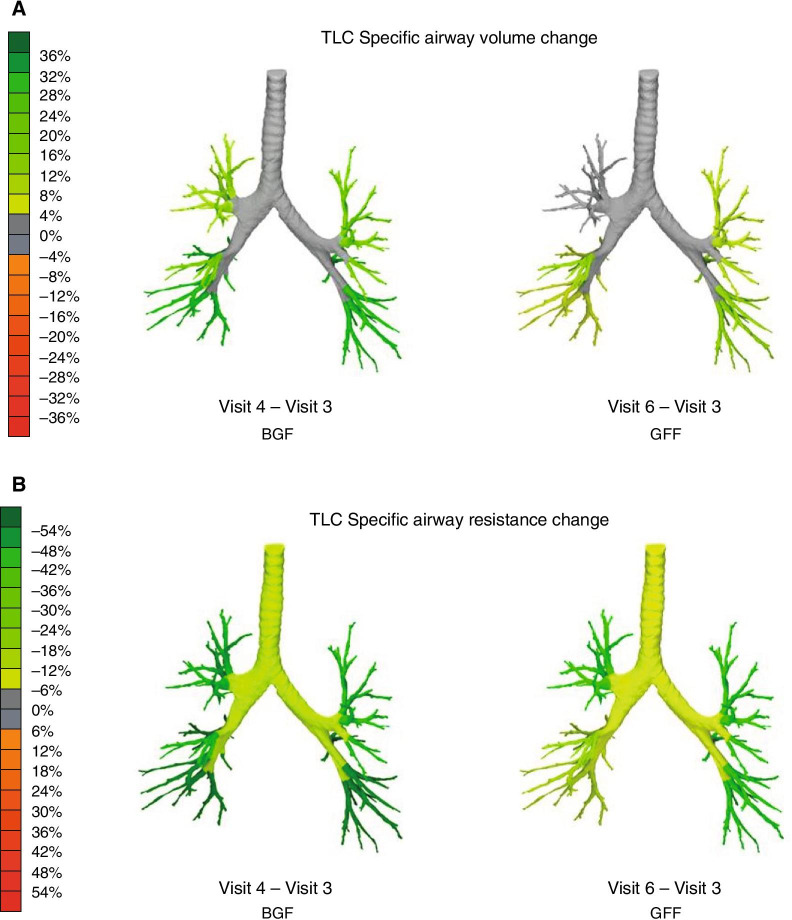
Percent change from baseline to Day 29 in **a** siVaw and **b** siRaw. Images show one representative patient’s data for siVaw (mL/L) and siRaw (kPa·s) percent change from baseline to Day 29 at TLC. Green coloring represents **a** an increase in airways volume and **b** a decrease in airway resistance. Orange coloring indicates the converse. *BGF* budesonide/glycopyrrolate/formoterol fumarate, *GFF* glycopyrrolate/formoterol fumarate, *siRaw* specific image-based airway resistance, *siVaw* specific image-based airway volume, *TLC* total lung capacity

BGF and GFF both statistically significantly improved siVaw from baseline at Day 29 by 72% and 53%, respectively (*p* < 0.0001 for both comparisons) (Table 2, Fig. 2a). A greater increase in siVaw was observed on average, across all lobes at TLC for treatment with BGF versus GFF (9%; *p* = 0.0061) (Table 3). In addition, statistically significant reductions of 50% and 48% in siRaw were observed on average across all lobes for both treatments, for BGF and GFF, respectively; *p* < 0.0001 for both comparisons) (Table 2, Fig. 2b). For siVaw, the difference between BGF and GFF was consistently observed over all lobes and by lobe, with a 9% overall difference corresponding to a 5–15% difference in each lobe; however, for siRaw, only a 3% overall difference corresponding to a 0–6% difference per lobe was observed at TLC for treatment with BGF versus GFF (*p* = 0.6094) (Table 3). Representative images from one patient are shown in Fig. 3a for siVaw and Fig. 3b for siRaw. Similar findings were observed in the mITT set. Sensitivity analyses based on trimmed data for siVaw and siRaw were consistent with the primary analyses (Additional file 1: Table A1).

**Table 2:** Baseline comparison for primary and secondary efficacy endpoints at Day 29 (ITT population)

	BGF 320/18/9.6 µg (*N* = 22)	GFF 18/9.6 µg (*N* = 23)
**Primary FRI endpoints**		
Untrimmed siVaw at TLC^a^		
Geometric mean, mL/L	2.05	2.00
Ratio to baseline (95% CI)	1.72 (1.38, 2.13)****	1.53 (1.28, 1.83)****
Untrimmed siRaw at TLC^a^		
Geometric mean, kPa·s	0.21	0.20
Ratio to baseline (95% CI)	0.50 (0.39, 0.63)****	0.52 (0.40, 0.67)****
**Secondary endpoints**		
* FRI*		
iVaw at TLC^a^		
Geometric mean, mL	2.74	2.71
Ratio to baseline (95% CI)	1.70 (1.37, 2.11)****	1.51 (1.26, 1.80)****
iRaw at TLC^a^		
Geometric mean, kPa·s/L	0.18	0.16
Ratio to baseline (95% CI)	0.50 (0.40, 0.63)****	0.52 (0.40, 0.68)****
*Spirometry*		
Post-dose FEV_1_^b^		
Mean change from baseline (95% CI)_,_ mL	346 (182, 509)***	273 (140, 405)***
*Body plethysmography*		
FRC^c^		
Mean change from baseline (95% CI), mL	–280 (–770, 210)	–500 (–810, –180)**
**Other endpoints**		
FVC^b^		
Mean change from baseline (95% CI), mL	422 (180, 663)**	302 (119, 485)**
FEF_25–75_^b^		
Mean change from baseline (95% CI), mL/s	263 (17, 509)*	83 (–153, 319)

^a^Based on *n* = 20 for BGF and *n* = 21 for GFF

^b^Based on *n* = 21 for BGF and for GFF

^c^Based on *n* = 22 for BGF and *n* = 21 for GFF

*p < 0.05; **p < 0.01; ***p < 0.001; ****p ≤ 0.0001

*BGF* budesonide/glycopyrrolate/formoterol fumarate, *CI* confidence interval, *FEF*_*25–75*_ forced expiratory flow 25–75% of FVC, *FEV*_*1*_ forced expiratory volume in 1 s, *FRC* functional residual capacity, *FRI* functional respiratory imaging, *FVC* forced vital capacity, *GFF* glycopyrrolate/formoterol fumarate, *iRaw* image-based airway resistance, *ITT* intent-to-treat, *iVaw* image-based airway volume, *siRaw* specific image-based airway resistance, *siVaw* specific image-based airway volume, *TLC* total lung capacity

**Table 3:** Treatment comparison for primary and secondary efficacy endpoints at Day 29 (ITT population)

	BGF 320/18/9.6 µg (*N* = 22)	GFF 18/9.6 µg (*N* = 23)
**Primary FRI endpoints**		
Untrimmed siVaw at TLC^a^		
Geometric LSM, mL/L	2.04	1.87
LSM ratio, BGF versus GFF (95% CI)	1.09 (1.03, 1.16)**	
Untrimmed siRaw at TLC^a^		
Geometric LSM, kPa·s	0.18	0.19
LSM ratio, BGF versus GFF (95% CI)	0.97 (0.85, 1.10)	
**Secondary endpoints**		
*FRI*		
Untrimmed iVaw at TLC^a^		
Geometric LSM, mL	2.62	2.38
LSM ratio, BGF versus GFF (95% CI)	1.10 (1.03, 1.17)**	
iRaw at TLC^a^		
Geometric LSM, kPa s/L	0.14	0.15
LSM ratio, BGF versus GFF (95% CI)	0.96 (0.85, 1.09)	
Spirometry		
FEV_1_^b^		
LS mean change from baseline (SE), mL	341 (69)	282 (69)
LSM difference, BGF versus GFF (95% CI)	60 (–14, 133)	
*Body plethysmography*		
FRC, mL (95% CI)^c^		
LS mean change from baseline (SE)	–310 (140)	–460 (142)
LSM difference, BGF versus GFF (95% CI)	150 (–230, 530)	
**Other endpoints**		
FVC^c^		
LS mean change from baseline (SE), mL	393 (94)	299 (95)
LSM difference, BGF versus GFF (95% CI)	94 (− 70, 259)	
FEF_25–75_^c^		
LS mean change from baseline (SE), mL/s	221 (102)	120 (103)
LSM difference, BGF versus GFF (95% CI)	101 (− 47, 250)	

^a^Based on n = 20 for BGF and n = 21 for GFF

^b^Based on *n* = 21 for BGF and for GFF

^c^Based on *n* = 22 for BGF and *n* = 21 for GFF

*p < 0.05; **p < 0.01; ***p < 0.001

*BGF* budesonide/glycopyrrolate/formoterol fumarate, *CI* confidence interval, *FEF*_*25–75*_ forced expiratory flow 25–75%, *FEV*_*1*_ forced expiratory volume in 1 s, *FRC* functional residual capacity, *FRI* functional respiratory imaging, *FVC* forced vital capacity, *GFF* glycopyrrolate/formoterol fumarate, *iRaw* image-based airway resistance, *ITT* intent-to-treat, *iVaw* image-based airway volume, *LSM* least squares mean, *SE* standard error, *siRaw* specific image-based airway resistance, *siVaw* specific image-based airway volume, *TLC* total lung capacity

Results for the secondary endpoints of iVaw and iRaw were consistent with the results for primary endpoints: statistically significant differences from baseline were observed for BGF and GFF on average, across lobes for iVaw (70%, *p* < 0.0001; 51%, *p* = 0.0001) and iRaw (50% and 48%, respectively; *p* < 0.0001 for both comparisons) (Table 2). The improvement in iVaw was 10% higher for BGF than GFF (*p* = 0.0051) (Table 3), and a by lobe analysis similarly showed a reduction in iRaw with BGF versus GFF (*p* = 0.5346) (Table 3).

Of note, as a percentage of the emitted dose following two actuations (320 μg, 18 μg, and 9.6 μg of budesonide, glycopyrrolate, and formoterol fumarate, respectively), deposition as determined by computational fluid dynamics and formulation characteristics was 38.1% of budesonide, 40.5% of glycopyrrolate, and 39.8% of formoterol fumarate at TLC at Day 29.

### FEV_1_

Significant improvements (mean increase in milliliters, mL [95% confidence interval (CI)]; *p*-value) were also observed for change from baseline in post-dose FEV_1_ with BGF (346 mL [182, 509]; *p* = 0.0003) and GFF (273 mL, [140, 405]; *p* = 0.0004), respectively (Table 2). As shown in Table 3, the differences between treatments in post-dose FEV_1_ did not reach statistical significance (least square [LS] mean difference [95% CI]: 60 mL [− 14, 133] *p* = 0.1057).

### FRC

A numerical improvement (mean decrease in milliliters, mL [95% CI]; *p*-value) from baseline was observed for FRC with BGF (− 280 mL, *p* = 0.2515) and a statistically significant improvement with GFF (− 500 mL, *p* = 0.0040), (LS mean difference [95% CI]: 150 mL [− 230, 530] *p* = 0.4256) (Table 3).

As mean changes can be influenced by outliers for a variable endpoint such as FRC in a small sample size like the one presented here, it should be noted that using median changes, a numerically larger decrease versus baseline was observed for BGF (− 360 mL) versus GFF (− 260 mL), respectively.

### FVC

Significant improvements (mean increase in milliliters, mL [95% CI]; *p*-value) in FVC were observed following treatment with BGF (422 mL [180, 663]; *p* = 0.0016) and GFF (302 mL [119, 485]; *p* = 0.0026) (Table 2), with the estimated difference between treatment arms of 94 mL (95% CI [− 70, 259], *p* = 0.2447; Table 3).

### FEF_25–75_

Following treatment with BGF, significant mean [95% CI] improvement in FEF_25–75_ of 263 mL/s ([17, 509]; *p* = 0.0374) was observed and following treatment with GFF, a numerical mean [95% CI] increase from baseline in FEF_25–75_ of 83 mL/s ([−153, 319]; *p* = 0.4710) was observed (Table 2), with the estimated difference between treatment arms in FEF_25–75_ of 101 mL (95% CI [− 47, 250], *p* = 0.1702; Table 3).

### Safety

Four (18.2%) and six patients (26.1%) experienced any AE in the BGF and GFF treatment periods, respectively (Table 4). One patient (4.5%) and three patients (13.0%) reported AEs related to BGF and GFF treatment, respectively (aphonia and bronchiolitis, dyspnea, and pruritus, respectively). In addition, one patient experienced a serious AE of colon cancer on BGF treatment, and one patient reported an AE of dyspnea leading to the discontinuation of study treatment while on GFF. Overall, BGF and GFF were well-tolerated and no new or unexpected AEs were reported. Safety findings were consistent with the known safety profiles of both treatments in patients with moderate-to-severe COPD.

**Table 4:** Overall summary of AEs, safety analysis set^a^

	BGF	GFF
	320/18/9.6 µg	18/9.6 µg
	(*N* = 22)	(*N* = 23)
Any AEs, *n* (%)	4 (18.2)	6 (26.1)
Allergy to arthropod sting	0	1 (4.3)
Aphonia	1 (4.5)	0
Bronchiolitis	0	1 (4.3)
Colon cancer	1 (4.5)	0
Cough	1 (4.5)	0
Dyspnea	0	2 (8.7)
Edema peripheral	0	1 (4.3)
Hypertension	1 (4.5)	0
Pruritus	0	1 (4.3)
Any AEs with an outcome of death, *n* (%)	0	0
Any serious TEAEs (including events with outcome of death), *n* (%)	1 (4.5)	0
Colon cancer	1 (4.5)	0
Any AE leading to discontinuation of study treatment, *n* (%)	0	1 (4.3)
Dyspnea	0	1 (4.3)
Any AEs related to study treatment^b^, *n* (%)	1 (4.5)	3 (13.0)
Aphonia	1 (4.5)	0
Bronchiolitis	0	1 (4.3)
Dyspnea	0	1 (4.3)
Pruritus	0	1 (4.3)
Any serious AEs related to study treatment^b^, *n* (%)	0	0

^a^Patients with multiple events in the same category were counted only once in that category. Patients with events in more than one category were counted once in each of those categories

^b^Investigator assessed

*AE* adverse event, *BGF* budesonide/glycopyrrolate/formoterol fumarate, *GFF* glycopyrrolate/formoterol fumarate, *TEAE* treatment-emergent adverse event

## Discussion

In this randomized, double-blind, Phase IIIb, crossover study, treatment with BGF and GFF demonstrated clinically meaningful and statistically significant improvements in airway volume and airway resistance in patients with moderate-to-severe COPD. The benefit of the addition of ICS to LAMA/LABA was demonstrated by the greater improvements observed in patients following treatment with BGF relative to GFF treatment.

Prior studies have found that both open [17] and fixed-dose [18] triple combination therapies significantly improve lung function, as assessed by spirometry, versus dual therapies in patients with moderate-to-very severe COPD [2, 3]. However, traditional spirometric measures are not able to evaluate regional differences in lung function and require large numbers of patients to assess the effects of ICS/LAMA/LABA due to the variability of traditional lung function measures [19]. FRI has been shown to be more sensitive to local lung function changes than spirometric measurements, indicating statistically significant small airway changes can occur without statistically significant changes in spirometric or body plethysmographic parameters [10]. Therefore, as shown in the current study, and in previously published studies [12, 13], fewer patients may be required to assess outcomes when using FRI measures. In addition, FRI can indirectly measure drug deposition in the airways [9].

The spirometry and body plethysmography results were directionally consistent with FRI results; however, no statistically significant differences were shown between BGF and GFF in any spirometry or plethysmography endpoint, indicating the increased sensitivity of the FRI parameters to detect differences between treatments in a small number of patients.

Prior results from scintigraphy studies showed BGF was homogeneously deposited throughout the airways of healthy subjects, with mass-deposition of 34.5% and 37.7% for 3- and 10-s breath-holds, respectively [4]. Additionally, Usmani et al. [5] showed that homogeneous lung deposition of BGF was achieved in patients with moderate-to-severe COPD, with a mass-deposition percentage of 32.1% recorded. In this study, FRI enabled detection of increasing airway volume and reducing airway resistance by addition of budesonide to LAMA/LABA inhaled therapy, after only 4 weeks of treatment. The improvements observed across all lobes of the lung using FRI in this study are consistent with the observed deposition throughout the entire lung in scintigraphy studies. FRI techniques estimated BGF mass-deposition to be approximately 38.1–40.5% of each monocomponent, correlating with the values observed within the BGF deposition study in healthy subjects [4], with the local benefit of ICS seen in patients with COPD [5] confirmed by these results. The in silico deposition reported by Usmani et al. [20] for beclomethasone dipropionate/formoterol fumarate/glycopyrronium pressurized MDI was 35.9%. Furthermore, in healthy subjects, the central to peripheral deposition ratios of BGF for 3- and 10-s breath-hold versus beclomethasone dipropionate/formoterol fumarate dry powder inhaler, were 0.56 and 0.72 versus 1.23, respectively [4, 21]. In patients with COPD, the central to peripheral deposition ratio for BGF was 0.9 [5]. The results of this study complement the effects observed in other endpoints, including symptom improvement and exacerbation frequency reduction.

Use of the ICS/LAMA/LABA triple, fixed-dose combination BGF 320/18/9.6 µg delivered via single Aerosphere inhaler was associated with an improvement in untrimmed siVaw by approximately 72% (increase), and in untrimmed siRaw by approximately 50% (decrease), reflecting the presence of improved bronchodilation/constriction. The LAMA/LABA treatment, GFF, was associated with 53% and 48% improvement in untrimmed siVaw and siRaw, respectively. The significant increase in airway volume (9%) of BGF versus GFF further supports the benefit of budesonide in patients with moderate-to-severe COPD versus LAMA/LABA [3, 22]. The smaller benefit of BGF in reducing of airway resistance relative to GFF (3%), could indicate a plateau effect. When an airway is dilated, lumen widening is observed with more airways being visible due to the CT scanner detecting branches that were previously too small. For siVaw, both widening and recruitment strengthen the signal; for siRaw, resistance decreases due to the widening, but increases again due to the recruitment (wider tubes mean lower resistance, but longer tubes increase resistance). Moreover, lung pathology may contribute to the fact that patients with COPD have increased expiratory airflow resistance caused by the loss of the alveolar attachments that usually maintain small airway shape. In addition, relaxation of the airways using bronchodilators will be limited by physiological effects of COPD such as increased secretions, lung hyperinflation and air trapping [23].

Budesonide is a glucocorticoid receptor agonist [24], which has been shown to reduce inflammatory biomarker levels in the lung [25–27]. Furthermore, when administered in combination with the LABA formoterol, budesonide inhibits formoterol-induced reductions in plasma membrane β_2_-receptors in the lung [28], possibly facilitating LABA-induced bronchodilation. Additionally, LABAs may prolong the anti-inflammatory effects of ICSs by increasing translocation of the glucocorticoid receptors following binding of an ICS and increasing the duration of receptors residence within the nucleus [29]. Thus, the differential FRI effects of triple therapy (ICS/LAMA/LABA) versus dual therapy (LAMA/LABA) on siVaw and siRaw in the current study could be attributable to a combination of reduced inflammation and enhanced bronchodilation.

One limitation of the study is that it only recruited patients not currently being treated with an ICS, all of whom had eosinophil counts of > 150 cells/mm^3^. The small number of patients in this study could also be considered a limitation in terms of assessing spirometry and body plethysmography endpoints. However, the number of patients within this study is consistent with that of prior FRI studies in similar populations [12, 13]. Finally, it should be acknowledged that these data are specific to BGF versus GFF and may not be generalizable to all ICS-containing triple therapy formulations.

## Conclusions

BGF and GFF were effective in increasing airway volume and decreasing airway resistance in patients with moderate-to-severe COPD. Importantly, the ICS component of BGF resulted in significantly greater increases in airway volume, as assessed using FRI, compared with GFF. The finding that BGF has effects on increasing airway volume and decreasing airway resistance throughout all lobes of the lung complements recent scintigraphy findings which show that BGF is also deposited throughout the large and small airways of the lung [5].

## Supplementary Information

**Additional file 1.** **Table A1.** Sensitivity analyses of co-primary endpoints based on trimmed data for siVaw and siRaw at TLC at Day 29 (ITT population).

## Data Availability

Data underlying the findings described in this manuscript may be obtained in accordance with AstraZeneca’s data sharing policy described at https://astrazenecagrouptrials.pharmacm.com/ST/Submission/Disclosure.

## Abbreviations

AE: Adverse event
BFF: Budesonide/formoterol fumarate
BGF: Budesonide/glycopyrrolate/formoterol fumarate
CI: Confidence interval
COPD: Chronic obstructive pulmonary disease
CT: Computer tomography
FEF_25–75_: Forced expiratory flow 25–75%
FEV_1_: Forced expiratory volume in 1 s
FRC: Functional residual capacity
FRI: Functional respiratory imaging
FVC: Forced vital capacity
GM: Geometric mean
GOLD: The Global Initiative for Chronic Obstructive Lung Disease
GFF: Glycopyrrolate/formoterol fumarate
HRCT: High-resolution computed tomography
ICS: Inhaled corticosteroids
iRaw: Image-based airway resistance
ITT: Intent-to-treat
iVaw: Image-based airway volume
LABA: Long-acting β_2_-agonists
LAMA: Long-acting muscarinic antagonists
LS: Least square
MDI: Metered dose inhaler
mITT: Modified intent-to-treat
SAE: Serious adverse event
SD: Standard deviation
siRaw: Specific image-based airway resistance
siVaw: Specific image-based airway volume
TLC: Total lung capacity
