# Supplementary material for: Functional respiratory imaging assessment of budesonide/glycopyrrolate/formoterol fumarate and glycopyrrolate/formoterol fumarate metered dose inhalers in patients with COPD: the value of inhaled corticosteroids
Source: Respir Res. 2021 Jul 1;22:191. doi: 10.1186/s12931-021-01772-2 (PMC8247252; doi:10.1186/s12931-021-01772-2)
Supplement: Supplementary file 1 — Additional file 1. Table A1. Sensitivity analyses of co-primary endpoints based on trimmed data for siVaw and siRaw at TLC at Day 29 (ITT population). [file 12931_2021_1772_MOESM1_ESM.docx]

**Supplementary material**

**Additional file 1: Table A1** Sensitivity analyses of co-primary endpoints based on trimmed data for siVaw and siRaw at TLC at Day 29 (ITT population)

|  | **BGF  320/18/9.6 µg (*N* = 22)** | **GFF 18/9.6 µg (*N* = 23)** |
| --- | --- | --- |
| **Co-primary FRI endpoints** |  |  |
| siVaw at TLC, mL/L (trimmed values)  Geometric mean  Ratio to baseline (95% CI)  Comparison between treatments  Geometric LSM (95% CI) | 1.68 1.53 (1.32, 1.78)^****^  1.69 (1.30, 2.18) | 1.70  1.42 (1.27, 1.59)^****^  1.59 (1.23, 2.05) |
| LSM ratio, BGF versus GFF (95% CI) | 1.06 (1.01, 1.11)^**^ | |
| siRaw at TLC, kPa∙s (trimmed values)  Geometric mean  Ratio to baseline (95% CI)  Comparison between treatments  Geometric LSM (95% CI) | 0.11 0.31 (0.20, 0.46)^****^  0.10 (0.07, 0.13) | 0.12  0.38 (0.27, 0.54)^****^  0.11 (0.08, 0.15) |
| LSM ratio, BGF versus GFF (95% CI) | 0.85 (0.74, 0.97)^*^ | |

^*^*p <* 0.05, ^**^*p ≤* 0.01, ^****^*p <* 0.0001

BGF, budesonide/glycopyrrolate/formoterol fumarate; CI, confidence interval; FRI, functional respiratory imaging; GFF, glycopyrrolate/formoterol fumarate; ITT, intent-to-treat; LSM, least squares mean; siRaw, specific image-based airway resistance; siVaw, specific image-based airway volume; TLC, total lung capacity
